# Comprehensive Evaluation of Vertical Sub-Surface Flow Constructed Wetlands with Aquatic Plants on Water Quality of Raw and Phyto-Remediated Poultry-Aquaculture Wastewater: A Principal Component Analysis

**DOI:** 10.3390/biology15110823

**Published:** 2026-05-23

**Authors:** Shadrach A. Akadiri, Pius O. O. Dada, Adekunle A. Badejo, Olayemi J. Adeosun, Oluwaseun T. Faloye, Oluwafemi E. Adeyeri, Laemthong Laokhongthavorn, Viroon Kamchoom

**Affiliations:** 1 Department of Agricultural and Biosystems Engineering, Federal University of Agriculture, Abeokuta 111101, Ogun State, Nigeria; 2 Department of Agriculture and Natural Resources, Ondo State Local Government Service Commission, Akure 340110, Ondo State, Nigeria; 3 Department of Civil Engineering, Federal University of Agriculture, Abeokuta 111101, Ogun State, Nigeria; 4 Department of Water Resources Management and Agrometeorology, Federal University Oye, Oye 370112, Ekiti State, Nigeria; faloye.temitope@gmail.com; 5 Department of Civil Engineering, Landmark University, Omu Aran 251103, Kwara State, Nigeria; 6 ARC Centre of Excellence for the Weather of the 21st Century, Fenner School of Environment and Society, The Australian National University, Canberra, 2600, Australia; 7 Excellent Centre for Green and Sustainable Infrastructure, School of Engineering, King Mongkut’s Institute of Technology Ladkrabang, Bangkok 10520, Thailand

**Keywords:** phytoremediation, constructed wetland, poultry-aquaculture wastewater, water quality index, heavy metals removal

## Abstract

This study investigated the efficiency of macrophyte-based phytoremediation systems using *Phragmites karka* and *Typha latifolia* for the treatment of poultry–aquaculture wastewater and its suitability for irrigation reuse. Physicochemical parameters, heavy metals, and water quality indices were analysed using correlation analysis and Principal Component Analysis (PCA). Strong positive correlations were observed among turbidity, nutrients, biochemical oxygen demand (BOD_5_), and chemical oxygen demand (COD), while dissolved oxygen (DO) showed significant negative relationships, indicating organic pollution-driven oxygen depletion. Heavy metals exhibited strong intercorrelations, suggesting common anthropogenic sources and similar removal pathways. PCA results revealed that the first three principal components (PCs) explained over 95% of the total variance, with positive values recorded from the first PC highlighting organic load, nutrient enrichment, and metal interactions as dominant factors controlling wastewater quality. The negative values of factor loadings obtained in the second and third PCs confirmed the roles of sedimentation, adsorption, microbial activity, and plant uptake in pollutant removal. Water Quality Index (WQI) values decreased drastically from highly polluted levels (>3000) in raw wastewater to <1.0 after 21 days of treatment, indicating excellent water quality. Sodium Absorption Ratio (SAR) also declined significantly, confirming a low sodicity risk. Both macrophytes demonstrated high treatment efficiency, with *Typha latifolia* showing slightly improved sodium reduction. Overall, the study highlights macrophyte-based systems as sustainable, cost-effective solutions for wastewater treatment and safe agricultural reuse.

## Simple Summary

The study showed that *Phragmites karka* and *Typha latifolia* effectively improved poultry–aquaculture wastewater quality. Strong correlations among pollutants indicated a common source, mainly organic and nutrient-rich waste, while dissolved oxygen decreased with higher pollution levels. Principal Component Analysis revealed that a few key factors controlled most water quality changes, depending on the type of macrophyte used. Both plants significantly reduced contaminants, including heavy metals, through natural processes like filtration and uptake. Water Quality Index values dropped from highly polluted to excellent after 21 days of treatment. Sodium levels also decreased, indicating the treated water is safe for irrigation and suitable for sustainable agricultural reuse when only chemical properties were considered and unsafe for irrigation when a holistic evaluation of the water quality was considered through the incorporation of the heavy metal values.

## 1. Introduction

The rapid expansion of integrated poultry–aquaculture systems has significantly increased the generation of nutrient-rich wastewater, posing serious environmental and public health concerns [1]. Such wastewater is typically characterised by high concentrations of organic matter, suspended solids, nutrients (nitrogen and phosphorus), and potentially toxic heavy metals derived from feed additives, veterinary inputs, and agricultural runoff [2,3]. When discharged untreated, these contaminants can degrade water quality, deplete dissolved oxygen, and disrupt aquatic ecosystems [4]. Consequently, the development of sustainable and cost-effective wastewater treatment technologies has become a global priority, particularly in regions facing water scarcity and increasing demand for agricultural reuse.

Constructed wetlands employing aquatic macrophytes have emerged as promising nature-based solutions for wastewater treatment [5,6]. These systems utilise plants, substrates, and associated microbial communities to remove pollutants through a combination of physical filtration, chemical adsorption, and biological transformation processes. Macrophytes such as the *Azolla* plant have been extensively tested and evaluated in the study location for heavy metal and other nutrient removal from polluted water [1,2]. But studies that have comprehensively evaluated the efficacy of *Phragmites karka* and *Typha latifolia* in removing pollutants from wastewater are still scarce. While constructed wetlands are widely recognised as cost-effective and environmentally sustainable, they are often constrained by long hydraulic retention times, variable treatment efficiency due to seasonal fluctuations, and limited performance in removing certain contaminants, such as heavy metals and emerging pollutants [6]. These limitations underscore the need for optimising plant selection and system design. These macrophytes were chosen due to their high biomass productivity, extensive root systems, tolerance to polluted environments, and well-documented capacity to enhance microbial activity in the rhizosphere. Compared to other commonly used species such as *Eichhornia crassipes* (water hyacinth) and *Lemna minor* (duckweed), emergent macrophytes like *Phragmites* and *Typha* offer greater structural stability, deeper root penetration, and improved oxygen transfer, which are critical for sustained pollutant removal [6]. Furthermore, the revised Introduction now includes a comparative perspective, noting that while floating macrophytes often demonstrate rapid nutrient uptake, they may be less effective in long-term treatment systems due to harvesting requirements and sensitivity to environmental changes. In contrast, emergent macrophytes provide more stable and efficient treatment over extended periods, particularly for integrated wastewater systems containing both organic and inorganic pollutants.

Recently, Akadiri et al. [7] reported the removal efficiency of pollutants in poultry wastewater using these macrophyte plants. But the heavy metals that are mostly influenced by phytoremediation when the two macrophyte plants (*Phragmites karka* and *Typha latifolia*) were used separately have not been reported; hence, the essence of applying the principal component analysis. It is unclear to date whether the removal of a heavy metal is dependent on the choice of the macrophyte plant. The macrophyte plants (*Phragmites karka* and *Typha latifolia*) were selected due to the fact that they are widely recognised for their high biomass production, extensive root systems, and tolerance to polluted environments, making them suitable candidates for phytoremediation applications [7,8,9]. Their rhizosphere provides a conducive environment for microbial activity, which plays a crucial role in the degradation of organic matter and nutrient cycling.

In addition to conventional physicochemical assessment, multivariate statistical techniques such as correlation analysis and Principal Component Analysis (PCA) have been increasingly applied to evaluate complex environmental datasets. But to date, these techniques have been scarcely applied in phytoremediation studies. These approaches enable the identification of relationships among variables, pollution sources, and dominant processes influencing water quality [10]. Furthermore, water quality indices, including the Water Quality Index (WQI) and sodium-related parameters, are essential tools for assessing the suitability of treated wastewater for irrigation purposes. Elevated sodium levels, for instance, can adversely affect soil structure and crop productivity, emphasising the need for careful evaluation before reuse [11]. Previous studies assessed the suitability of raw aquaculture wastewater for irrigation purposes by only considering the physico-chemical properties [12,13] and neglecting heavy metals, or by comparing each parameter with the international standard [14,15]. In most cases, some of these parameters are out of range when compared with the international standard, which could make the judgment for human use confusing. An alternative to solve this problem of misjudgment is through the use of the water quality index [16].

Despite the growing body of research on constructed wetlands, there remains a need for comprehensive studies that integrate physicochemical characterisation, heavy metal dynamics, multivariate statistical analysis, and irrigation suitability assessment within a single framework. This is particularly important for poultry–aquaculture wastewater, which presents unique challenges due to its combined organic and inorganic pollutant load. Therefore, this study aims to evaluate the performance of *Phragmites karka* and *Typha latifolia* in the treatment of poultry–aquaculture wastewater. Specifically, the study (i) examines the relationships among physicochemical parameters and heavy metals, (ii) studies and identifies the dominant factors controlling wastewater quality through PCA, and (iii) assesses the suitability of the treated effluent for irrigation using water quality indices by considering physicochemical and heavy metals for its evaluation. The findings are expected to contribute to the development of sustainable wastewater management strategies and promote safe water reuse in agricultural systems.

## 2. Materials and Method

### 2.1. Study Area and Experimental Design

The study was conducted at the Centre of Excellence in Agriculture Development and Sustainable Environment (CEADESE), located within the Federal University of Agriculture in Abeokuta, Ogun State, Nigeria. The location is characterised by wet and dry seasons. The rainy season starts in Mid-March and ends in late October, while the dry season starts in November and ends in March. The experiment encompasses an experimental phytoremediation setup designed to evaluate the treatment efficiency of two emergent macrophytes, *Phragmites karka* and *Typha latifolia*, for poultry–aquaculture wastewater. Wastewater was sourced from an integrated poultry–aquaculture system and transported to the experimental units for treatment. The system consisted of constructed wetland containers operated under controlled conditions (control valves were connected to each constructed wetland and were not opened until the 7-, 14- and 21-day retention periods were attained), with defined hydraulic retention periods of 7, 14, and 21 days. Each treatment unit was planted separately with either *Phragmites karka* (PT) or *Typha latifolia* (TT), while raw wastewater served as the control.

The vertical sub-surface flow constructed wetland (VSFCW) was adopted with filter media arranged in the container. The media contains gravel and sand with about 20 cm thickness each in the container. The capacity of the container was 105 L, with a 0.6 m diameter and a 0.5 m height. In the design of the constructed wetland, factors such as flow rate, aspect ratio (length-to-width ratio), hydraulic retention time, and hydraulic loading rate were considered to improve the performance of the system [17,18]. The macrophyte, *Phragmites karka*, was established using an asexual propagation method, while the method of establishing *Typha latifolia* was by cuttings, as obtained from mature plants collected from a nearby stream. The experimental design is such that each treatment consisted of a constructed wetland with poultry-aquaculture wastewater treated with *Phragmites karka* and poultry-aquaculture wastewater treated with *Typha latifolia*, replicated nine times as shown in Figure S1, while the raw wastewater serves as the control treatment. The density was nine plants per container, similar to the research by Chang et al. [19]. The Wastewater was allowed to enter the constructed wetland by upflow as illustrated in Figures S1 and S2 (Supplementary Data).

### 2.2. Sample Collection and Analysis

Wastewater samples were collected at each retention interval (0, 7, 14, and 21 days) across three seasonal periods (November–January, March–May, and July–September). The undiluted samples of the poultry-aquaculture wastewater were collected directly from the storage reservoir before their introduction to the vertical subsurface flow constructed wetlands (VSFCW). The constructed wetland was nine containers for each macrophyte, meaning nine replications. In each season, samples of the treated wastewater were collected from the wetlands based on the nine replications, after retention periods of 7, 14, and 21 days had been attained. The raw wastewater and those samples collected after treatment at 7, 14, and 21 days of retention times were gathered and placed in a pristine 75 cL container, with the grab sampling method adopted. This approach was adopted in all three seasons. In each season, the samples were preserved at a temperature of 4 °C until they were transported to the laboratory for the required analysis. Samples were preserved and analysed following standard procedures recommended by the American Public Health Association [20]. Physicochemical parameters, including colour, turbidity, pH, dissolved oxygen (DO), chloride (Cl^−^), nitrate (NO_3_^−^), sulphate (SO_4_^2−^), biochemical oxygen demand (BOD_5_), and chemical oxygen demand (COD) were determined using standard analytical methods. The physico-chemical properties of treated and untreated wastewater were determined according to the method reported in our previously published papers [7,21], as presented in Table 1.

### 2.3. Heavy Metal Analysis

Heavy metals, including arsenic (As), cadmium (Cd), copper (Cu), chromium (Cr), iron (Fe), lead (Pb), manganese (Mn), zinc (Zn), and nickel (Ni), were analysed using atomic absorption spectrophotometry after appropriate digestion of the samples using concentrated HNO_3_ (Table 1). Quality control was ensured through the use of blanks, standards, and replicate analyses.

### 2.4. Water Quality Assessment

The suitability of treated wastewater for irrigation was evaluated using the Water Quality Index (WQI) and sodium-related indices. The WQI was calculated using the weighted arithmetic method (Equation (1)), incorporating multiple physicochemical parameters to provide a single measure of overall water quality [24]

(1) $$\mathrm{WQI}=\frac{{\sum (Q}_{i}W_{i})}{{\sum (W}_{i})}$$

where Q_i_ is the water quality rating of the ith water quality parameter, W_i_ is the unit weight of the parameter water quality at the ith rating. The water quality rating, Q_i_, was calculated using Equation (2)

(2) $$Q_{i}=\frac{100\;(V_{i}-V_{0})}{S_{i}-V_{0}}$$

where V_i_ is the actual amount of the measured parameter, V_0_ stands for the ideal value of the parameter (V_0_ = 0), except for the pH and DO, which are expected to be = 7 and 14.6 mg/L, respectively. The S_i_ represents the standard allowable value for the ith parameter. The allowable limits according to the irrigation standard and the international standard [11,25] are illustrated in Table 2 below. The unit weight (W_i_) is calculated using Equation (3). The term K is a proportional constant and is calculated using Equation (3). According to Makumbura et al. [24]. WQI > 100 is unsuitable for agricultural purposes, including irrigation, while WQI < 100 indicate water suitable for irrigation

(3) $$W_{i}=\frac{K}{S_{i}};\;K=\frac{1}{\sum \left(\frac{1}{S_{i}}\right)}$$

The Sodium Absorption Ratio (SAR) and sodium percentage (Na %) were calculated to assess potential sodicity hazards to soil, following guidelines provided by Meireles et al. [26]. The SAR was calculated using Equation (4), as given below

(4) $$\mathrm{SAR}=\frac{{Na}^{+}}{\sqrt{\frac{{Mg}^{2+}+{Ca}^{2+}}{2}}}$$

**Table 2:** International standards for the assessment of water quality for irrigation.

Parameter	International Standard
Colour (HU)	5.13
Turbidity (NTU)	10
pH	6.50–8.50
DO (mg/L)	6.4
Cl^−^ (mg/L)	350
NO_3_^−^ (mg/L)	50
SO_4_^2−^ (mg/L)	500
BOD_5_ (mg/L)	4
COD (mg/L)	30
Arsenic (As) (mg/L)	0.25
Cadmium (Cd) (mg/L)	0.003
Copper (Cu) (mg/L)	1
Chromium (Cr) (mg/L)	0.05
Iron (Fe) (mg/L)	0.5
Lead (Pb) (mg/L)	0.1
Manganese (Mn) (mg/L)	1
Zinc (Zn) (mg/L)	2.4
Nickel (Ni) (mg/L)	0.02

**Sources:** Pescod [27]. **Note:** HU means Hazen Unit, NTU means Nephelometric Turbidity Unit.

### 2.5. Statistical Analysis

One-Way Analysis of Variance (ANOVA) was carried out to separate the means of the data at 5% level of significance using Tukey’s test. Descriptive statistics were used to summarise the data, and the data were presented as mean ± standard deviation. Pearson correlation analysis was employed to evaluate relationships among physicochemical parameters and heavy metals. Correlation analysis is an important preliminary statistical tool that helps to identify relationships among physicochemical parameters and contaminants, thereby providing insight into possible common sources, co-mobility, and similar transformation pathways. The correlational analysis is also important to be carried out before Principal Component Analysis (PCA) to reveal the extent of collinearity that exists among parameters. Principal Component Analysis (PCA) was applied to identify the major factors influencing water quality, as a result of the water source and phyto-remediation, and to reduce data dimensionality. Eigenvalues greater than one were considered significant based on the Kaiser criterion [28], and factor loadings greater than ±0.50 were interpreted as strong contributions. Data across all seasons were considered under each macrophyte separately, in order to observe the variation effectively for the physico-chemical and heavy metals. This approach enables the identification of the factors that control water quality through PCA.

All statistical analyses were performed using Minitab version 18.0 for the ANOVA and PCA, while the SPSS version 27 was used to assess the suitability of the dataset for PCA analysis by determining the Kaiser–Meyer–Olkin (KMO) and Bartlett tests. The KMO produced values greater than 0.70 in all cases, while the Bartlett tests were less than 0.0001, thus indicating high suitability for PCA. The Varimax approach was used for the component rotation as described in Faloye [29,30]. The graphical representations, such as biplots, were used to visualise relationships among variables.

## 3. Results and Discussion

### 3.1. Raw Poultry-Aquaculture Wastewater Characterisation and the Impact of Phytoremediation on Its Physico-Chemical and Heavy Metal Properties

Tables S1–S4 (Supplementary Data) and the main dataset consistently show that both *Phragmites karka* and *Typha latifolia* significantly improved wastewater quality across all seasons and retention periods. In most cases, the differences in the effect of the macrophyte plants on the reduction of heavy metals and other nutrients were not significant (*p* > 0.05), but significant (*p* < 0.05) when compared to the raw wastewater. This implies that they remove the heavy metals similarly. This may be attributed to the structural similarities common to both plants, in terms of root system structure, which aid the absorption of the heavy metals [31]. Progressive reductions in turbidity, colour, BOD_5_, COD, nitrate, sulphate, and major ions (Na, K, Ca, Mg) were observed from 7 to 21 days of retention, accompanied by a substantial increase in DO. For instance, BOD_5_ decreased from 27.9 mg/L in raw wastewater to 2.33 mg/L after 21 days, while COD reduced from >53.7 mg/L to <4.29 mg/L. Turbidity and colour also declined markedly, indicating effective removal of suspended and dissolved solids. The increase in DO to values approaching 4–5 mg/L reflects improved oxygenation due to plant-mediated aeration and microbial activity in the rhizosphere. These findings are consistent with the known mechanisms of pollutant removal in constructed wetlands, including sedimentation, filtration, microbial degradation, and plant uptake [9]. Seasonal variation influenced treatment efficiency, with slightly enhanced performance observed during the July–September period, likely due to favourable temperatures (which may result in growth rate enhancement of the macrophytes) and increased biological activity [32,33]. Nevertheless, both macrophytes maintained consistent treatment efficiency across all seasons. The supplementary results (Tables S5–S7—Supplementary Data) show that heavy metals were significantly reduced with increasing retention time. Metals such as arsenic (As), cadmium (Cd), chromium (Cr), and lead (Pb) were reduced to non-detectable levels after 21 days, while others, such as copper (Cu), zinc (Zn), and iron (Fe), showed substantial reductions.

### 3.2. Relationship Among the Physico-Chemical Properties of the Poultry-Aquaculture Wastewater as Influenced by the Macrophyte Plants

Table 1 and Table 2 present the correlation relationships among the physicochemical parameters of poultry–aquaculture wastewater treated with *Phragmites karka* and *Typha latifolia*. The correlation coefficients indicate strong interactions among organic load indicators, nutrient concentrations, and dissolved oxygen, reflecting the effectiveness of macrophyte-based phytoremediation systems. In Table 3, colour shows very strong positive correlations with turbidity (r = 0.984), pH (r = 0.964), chloride (r = 0.924), nitrate (r = 0.951), sulphate (r = 0.965), BOD_5_ (r = 0.966), and COD (r = 0.968), all significant at *p* ≤ 0.05. Similarly, turbidity exhibits strong positive correlations with most chemical parameters, including nitrate (r = 0.972), sulphate (r = 0.981), BOD_5_ (r = 0.979), and COD (r = 0.980). These strong associations suggest that suspended solids and coloured compounds in the wastewater are closely linked with the organic and nutrient load of the effluent. Such relationships are typical in wastewater systems where particulate organic matter contributes simultaneously to turbidity, colour, and biochemical oxygen demand [9]. Table 4 demonstrates a similar pattern for wastewater treated with *Typha latifolia*. Colour and turbidity also show a strong positive correlation (r = 0.983), and both parameters correlate positively with nutrient and organic load indicators, such as nitrate, sulphate, BOD_5_, and COD. These relationships indicate that reductions in turbidity and colour during treatment are associated with simultaneous removal of organic matter and dissolved ions [34,35]. Macrophytes facilitate this removal through sedimentation, filtration by plant roots, and microbial degradation processes in the rhizosphere [28].

A notable pattern in both tables is the strong negative correlation between dissolved oxygen (DO) and most other parameters. In Table 3, DO shows negative correlations with colour (r = −0.956), turbidity (r = −0.974), nitrate (r = −0.991), BOD_5_ (r = −0.991), and COD (r = −0.991). Similarly, in Table 4, DO is negatively correlated with colour (r = −0.949), turbidity (r = −0.970), nitrate (r = −0.993), and BOD_5_ (r = −0.987). This inverse relationship indicates that higher organic and nutrient loads lead to increased microbial respiration and oxygen consumption, thereby reducing dissolved oxygen levels. Such patterns are widely reported in wastewater and aquatic ecosystems where organic pollution drives oxygen depletion [4]. Both tables show extremely strong positive correlations between BOD_5_ and COD (r = 0.998 in Table 3; r = 0.974 in Table 4), indicating that both parameters respond similarly to the concentration of biodegradable organic matter in the wastewater. The strong correlation between these two parameters confirms that the organic load in poultry–aquaculture wastewater is largely biodegradable. Similar strong relationships between BOD_5_ and COD have been observed in wastewater treated using constructed wetlands and macrophyte systems [21]. Nitrate, chloride, and sulphate also display strong positive correlations with turbidity, colour, and organic load parameters in both tables. This suggests that nutrient concentrations are associated with suspended and dissolved organic matter in the wastewater. Macrophytes such as *Phragmites karka* and *Typha latifolia* enhance nutrient removal through plant uptake, microbial nitrification–denitrification processes, and adsorption within the wetland substrate [36].

#### Comparative Implications of the Two Macrophyte Systems

Although both macrophyte systems show similar correlation trends, the slightly stronger correlations observed in the *Phragmites karka* system (Table 5) suggest a potentially stronger interaction between organic load and physicochemical parameters during treatment. This may be attributed to the extensive root system and high biomass production of *Phragmites* species, which enhance microbial activity and pollutant removal efficiency in constructed wetland systems [9]. However, *Typha latifolia* also demonstrates strong pollutant interactions and is widely recognised for its effectiveness in nutrient and organic matter removal in wastewater treatment wetlands. Overall, the correlation matrices indicate that both macrophyte species significantly influence the physicochemical dynamics of poultry–aquaculture wastewater, supporting their suitability for phytoremediation and eco-friendly wastewater management [37].

### 3.3. Relationship Among the Heavy Metals’ Concentrations of the Poultry-Aquaculture Wastewater as Influenced by the Macrophyte Plants

Table 5 and Table 6 present the correlation relationships among heavy metals in poultry–aquaculture wastewater treated using the macrophytes *Phragmites karka* and *Typha latifolia*. The correlation matrices show strong positive relationships among most heavy metals, indicating possible similarities in their sources, mobility, and removal mechanisms during phytoremediation treatment. Table 5 demonstrates strong and significant positive correlations among the heavy metals, with correlation coefficients ranging from 0.856 to 0.983 (*p* ≤ 0.05). For instance, arsenic (As) shows strong positive correlations with cadmium (Cd) (r = 0.909), copper (Cu) (r = 0.933), chromium (Cr) (r = 0.947), lead (Pb) (r = 0.951), manganese (Mn) (r = 0.911), zinc (Zn) (r = 0.956), and nickel (Ni) (r = 0.908). These relationships suggest that these metals likely originate from similar contamination sources within poultry–aquaculture wastewater systems, such as feed additives, veterinary drugs, and agricultural runoff. The strongest correlations observed in the matrix are between Mn and Pb (r = 0.983), as well as between Zn and Cr (r = 0.981). Such strong relationships indicate that these metals may behave similarly during treatment processes and could be removed simultaneously through adsorption, precipitation, and plant uptake mechanisms. Aquatic macrophytes such as *Phragmites karka* are known for their extensive root systems that enhance metal accumulation and immobilisation within wetland substrates and plant tissues [9]. Nickel (Ni) also exhibits strong correlations with several metals, including Cd (r = 0.974), Cr (r = 0.972), and Cu (r = 0.961). These relationships indicate that nickel removal during phytoremediation may occur through similar pathways as other transition metals, particularly through adsorption onto sediment particles and rhizosphere-mediated microbial processes. Constructed wetland systems dominated by macrophytes are widely recognised for their capacity to reduce heavy metal concentrations through a combination of phytoaccumulation and sedimentation [34].

The correlation pattern observed in Table 6 is largely consistent with that in Table 5, indicating that *Typha latifolia* also facilitates similar heavy metal removal mechanisms in poultry–aquaculture wastewater treatment. Strong positive correlations exist among most metals, including As–Cd (r = 0.909), Cu–Cr (r = 0.966), and Zn–Cr (r = 0.981). These strong correlations indicate that heavy metals tend to co-occur and may be influenced by similar environmental processes within the treatment system. Lead (Pb) also exhibits strong positive relationships with Cu (r = 0.972) and Cr (r = 0.963), suggesting a common origin and similar transport behaviour in wastewater. Metals such as Pb, Cu, and Zn are commonly associated with poultry production systems due to their presence in feed supplements and agricultural inputs. Their simultaneous reduction during treatment reflects the capacity of macrophytes and associated microbial communities to immobilise and accumulate these metals within plant tissues and wetland substrates [29]. The strong correlation between Cd and Ni (r = 0.974) further suggests that these metals respond similarly to phytoremediation processes. *Typha latifolia* is widely recognised as an efficient phytoremediator due to its high biomass production, extensive rhizome network, and ability to accumulate heavy metals within root and shoot tissues. These characteristics enhance the removal of toxic metals from wastewater systems through phytoextraction and rhizofiltration processes [9].

#### Implications for Phytoremediation Efficiency

The strong positive correlations among heavy metals observed in both Table 5 and Table 6 suggest that the contaminants likely originate from similar sources and respond similarly during treatment with macrophyte systems. The comparable correlation patterns in both *Phragmites karka* and *Typha latifolia* systems indicate that both plant species are effective in facilitating the removal or immobilisation of heavy metals in poultry–aquaculture wastewater. Macrophyte-based treatment systems enhance heavy metal removal through several mechanisms, including plant uptake, adsorption onto root surfaces, microbial transformations, and sedimentation within the wetland substrate. These processes collectively contribute to the reduction in toxic metal concentrations and improve overall wastewater quality. Constructed wetlands utilising emergent macrophytes are therefore considered sustainable and cost-effective technologies for treating agricultural wastewater contaminated with heavy metals. Harvested macrophytes (*Phragmites karka* and *Typha latifolia*) can be periodically removed and safely managed through controlled composting, anaerobic digestion, or incineration, depending on the level of metal accumulation [32]. For systems with elevated heavy metal content, thermal treatment (e.g., incineration) is recommended to prevent re-release into the environment, with ash disposed of in accordance with hazardous waste guidelines [32]. Regarding the filtration layer (gravel and sand), contaminants are largely immobilised through adsorption and precipitation processes, and the substrates can remain effective for extended periods. When saturation occurs, the material can be replaced and disposed of in controlled landfill systems or regenerated where feasible. Previous studies have demonstrated that constructed wetlands are environmentally safe when proper maintenance, periodic harvesting, and substrate management are implemented [35,36].

Overall, the correlation results highlight the potential of both *Phragmites karka* and *Typha latifolia* as suitable macrophytes for phytoremediation of heavy metal-contaminated poultry–aquaculture wastewater. In this study, strong positive correlations among parameters such as turbidity, nutrients, BOD_5_, and COD suggest that these pollutants are largely derived from the same organic loading and are removed through linked processes, including sedimentation, microbial degradation, and plant uptake. Similarly, strong correlations among heavy metals indicate shared anthropogenic sources and comparable geochemical behaviour.

### 3.4. Principal Component Analysis of the Physico-Chemical Properties of the Poultry-Aquaculture Wastewater as Affected by the Macrophyte Plantes

Principal Component Analysis (PCA) is widely used in environmental studies to reduce complex datasets and identify the major factors controlling water quality variations. In wastewater treatment systems, PCA helps reveal relationships among physicochemical parameters and determine the dominant processes influencing pollutant removal [38]. The PCA results presented in Table 7 and Table 8a,b provide insight into the underlying mechanisms controlling the physicochemical characteristics of poultry–aquaculture wastewater treated with *Phragmites karka* and *Typha latifolia*. Table 5 presents the eigenvalues and percentage variance explained by each principal component for both macrophyte systems. For the *Phragmites karka* treatment system, the first three principal components (PC1, PC2, and PC3) have eigenvalues of 3.0651, 2.9688, and 2.8342, explaining 34.1%, 33.0%, and 31.5% of the total variance, respectively. Together, these three components account for approximately 98.6% of the total variance, indicating that most of the variability in the physicochemical dataset can be explained by these principal components. This suggests that the wastewater characteristics are strongly controlled by a limited number of environmental factors.

In the *Typha latifolia* system, the first four principal components have eigenvalues of 3.1852, 2.5148, 1.6516, and 1.5587, explaining 35.4%, 27.9%, 18.4%, and 17.3% of the variance, respectively. These four components cumulatively explain about 99.0% of the total variance, indicating that multiple processes influence water quality changes in this treatment system. Eigenvalues greater than 1 are generally considered significant according to the Kaiser criterion and represent the dominant components controlling the dataset structure [35]. The high variance explained by the first few components in both macrophyte systems indicates strong interrelationships among physicochemical parameters, including turbidity, nutrients, and organic load indicators. Similar PCA patterns have been reported in constructed wetland studies, where a few dominant factors account for most of the variability in wastewater quality [9].

#### 3.4.1. Factor Loading Analysis for *Phragmites karka* Based on the Physico-Chemical Characterisation

Table 8a shows the factor loadings for the physicochemical variables in the *Phragmites karka* treatment system. Factor loadings represent the degree of correlation between each variable and the principal components. Generally, loadings greater than ±0.50 are considered strong and indicate a significant contribution of the variable to the component. In Factor 1, strong positive loadings are observed for turbidity (0.609), chloride (0.780), sulphate (0.571), BOD_5_ (0.573), COD (0.592), nitrate (0.528), and colour (0.523). These variables are commonly associated with organic pollution and dissolved solids in wastewater. Therefore, Factor 1 can be interpreted as an organic pollution and nutrient enrichment factor, reflecting the influence of suspended solids and organic matter derived from poultry and aquaculture waste inputs.

Dissolved oxygen (DO) shows a strong negative loading in Factor 1 (−0.586), indicating an inverse relationship with organic pollution parameters. This relationship is consistent with wastewater systems where higher organic loads increase microbial respiration, leading to oxygen depletion in aquatic environments. Similar relationships between DO and organic pollution indicators have been documented in wastewater treatment wetlands [28].

Factor 2 shows strong positive loading for DO (0.633) and negative loadings for nitrate (−0.682), BOD_5_ (−0.624), COD (−0.599), sulphate (−0.588), and pH (−0.596). This component likely represents biological oxidation processes occurring in the rhizosphere of the macrophytes. The oxygen released by plant roots enhances microbial degradation of organic matter and nutrient transformation processes such as nitrification and denitrification. Factor 3 also shows significant negative loadings for colour (−0.714), turbidity (−0.611), and pH (−0.640), suggesting that this component represents particulate matter removal and sedimentation processes facilitated by the dense root network of the macrophyte system [34,35].

#### 3.4.2. Factor Loading Analysis for *Typha latifolia* Based on the Physico-Chemical Characterisation

The factor loading pattern for the *Typha latifolia* system shows similar but slightly different characteristics (Table 8b). Factor 1 exhibits strong positive loadings for pH (0.788), nitrate (0.653), sulphate (0.636), BOD_5_ (0.600), turbidity (0.531), and colour (0.547), while DO shows a strong negative loading (−0.634). This component can therefore be interpreted as representing nutrient enrichment and organic pollution processes similar to those observed in the *Phragmites karka* system.

Factor 2 shows strong positive loadings for chloride (0.729), turbidity (0.550), nitrate (0.520), sulphate (0.520), and COD (0.519). This factor likely represents ionic composition and dissolved solids, which are influenced by wastewater inputs from poultry feed residues and aquaculture effluents. Factor 3 and Factor 4 show moderate negative loadings for several parameters, including colour, turbidity, BOD_5_, and COD, indicating secondary processes such as sedimentation, microbial degradation, and plant uptake. Macrophytes such as *Typha latifolia* are well known for their ability to enhance pollutant removal through root filtration and microbial [34,35].

### 3.5. Principal Component Analysis of the Heavy Metals’ Properties of the Poultry-Aquaculture Wastewater as Affected by the Macrophyte Plants

Principal Component Analysis (PCA) was applied to evaluate the relationships and controlling factors of heavy metals in poultry–aquaculture wastewater treated with *Phragmites karka* and *Typha latifolia*. This multivariate approach enables the identification of dominant pollution sources and the mechanisms governing metal distribution and removal in phytoremediation systems [25]. Table 9 presents the eigenvalues and percentage variance explained by each principal component for both macrophyte systems. In the *Phragmites karka* system, the first three principal components (PC1, PC2, and PC3) explain 33.7%, 31.5%, and 21.7% of the total variance, respectively, contributing a cumulative variance of approximately 86.9%. Inclusion of the fourth component increases the cumulative variance to about 96.9%, indicating that these components capture most of the variability in heavy metal distribution.

For the *Typha latifolia* system, the first three principal components explain 34.5%, 33.4%, and 28.6% of the variance, respectively, accounting for a cumulative variance of approximately 96.5%. This indicates that fewer components are required to explain the variability in this system, suggesting a more uniform behaviour of heavy metals during treatment. According to the Kaiser criterion (eigenvalues > 1), these components represent the dominant processes controlling heavy metal dynamics [25,38]. The high variance explained by the first few components in both systems indicates strong intercorrelations among the heavy metals, suggesting common sources and similar geochemical behaviour. Such patterns are typical in wastewater environments where metals originate from anthropogenic inputs such as feed additives, fertilisers, and aquaculture activities [9].

#### 3.5.1. Factor Loading Analysis for *Phragmites karka* Based on the Heavy Metals’ Characterisation

Table 10a shows the factor loadings of heavy metals in the *Phragmites karka* system. Factor 1 exhibits strong positive loadings for Mn (0.798), Pb (0.704), Cu (0.598), Zn (0.596), Cr (0.565), As (0.535), and Cd (0.433). This factor can be interpreted as a mixed anthropogenic pollution factor, representing metals introduced from similar sources such as poultry feed, manure, and aquaculture inputs. Factor 2 shows strong negative loadings for Cd (−0.769), Ni (−0.730), Cr (−0.604), and Cu (−0.532), indicating a metal mobility and transformation factor. This component likely reflects processes such as adsorption, precipitation, and redox reactions occurring in the wetland substrate and rhizosphere [39]. These processes influence the bioavailability and partitioning of metals between water, sediment, and plant tissues [39]. Factor 3 is dominated by a strong negative loading for Fe (−0.716), suggesting that iron plays a distinct role in controlling heavy metal behaviour. Iron oxides are known to act as important sorbents for heavy metals, facilitating their immobilisation in wetland systems through co-precipitation and adsorption mechanisms [28]. Overall, the factor structure indicates that *Phragmites karka* facilitates heavy metal removal through multiple interacting processes, including plant uptake, sedimentation, and chemical stabilisation.

#### 3.5.2. Factor Loading Analysis for *Typha latifolia* Based on the Heavy Metals’ Characterisation

The factor loading pattern for the *Typha latifolia* system shows both similarities and distinctions. Factor 1 exhibits strong positive loadings for Cd (0.766), Ni (0.768), Cr (0.659), Cu (0.596), and As (0.496), indicating a dominant contamination factor associated with anthropogenic inputs. Factor 2 shows strong negative loadings for Mn (−0.776), Pb (−0.743), Cu (−0.611), and Zn (−0.568), suggesting a metal retention and sequestration factor. This component likely reflects the efficiency of *Typha latifolia* in immobilising heavy metals within its root system and surrounding sediments. Macrophytes such as *Typha* species are known to accumulate metals in below-ground tissues, thereby reducing their mobility in aquatic systems [29]. Factor 3 is strongly influenced by Fe (−0.771) and Zn (−0.631), highlighting the role of iron-mediated processes in heavy metal immobilisation. Iron oxides and hydroxides in wetland sediments play a crucial role in binding heavy metals and reducing their bioavailability. Compared to *Phragmites karka*, the *Typha latifolia* system shows a slightly more distinct separation of factors, indicating clearer differentiation between contamination sources and removal mechanisms.

### 3.6. Water Quality Assessment of the Treated Poultry Aquaculture Wastewater for Irrigation

The assessment of water quality for irrigation suitability is a critical step in evaluating the effectiveness of phytoremediation systems. Table 11 and Table 12 present the weighted average Water Quality Index (WQI) and sodium-related indices for poultry–aquaculture wastewater treated with *Phragmites karka* (PT) and *Typha latifolia* (TT) across different seasons and retention periods. These parameters provide insight into the overall improvement in water quality and its suitability for agricultural reuse. The WQI values in Table 11 indicate a substantial improvement in water quality with increasing retention time for both macrophyte systems. This is evident in the significant (*p* < 0.05) improvement in the WQI with an increase in retention time in Table 11. The raw wastewater exhibits extremely high WQI values across all seasons (e.g., 4988.3 in November–December and 3732.6 in March–May), indicating very poor water quality unsuitable for irrigation, according to the FAO [11] standard (Supplementary Data: Table S8). High WQI values are typically associated with elevated levels of organic matter, nutrients, and suspended solids (Water Quality Index). After 7 days of treatment, there is a noticeable reduction in WQI, although the values remain high, suggesting partial removal of contaminants. By 14 days, a dramatic decline in WQI is observed, particularly in the July–September period, where values drop to as low as 7.53 (PT) and 7.27 (TT). This sharp reduction reflects significant removal of pollutants through processes such as sedimentation, microbial degradation, and plant uptake. At 21-day retention, WQI values fall below 1.0 in all seasons for both macrophytes (e.g., 0.43–0.37 in Nov–Dec and 0.39–0.39 in Jul–Sep), indicating excellent water quality suitable for irrigation purposes [11] (Supplementary Data: Table S8). This trend demonstrates that extended retention time enhances treatment efficiency and confirms the effectiveness of macrophyte-based systems in improving wastewater quality. Similar improvements in WQI have been reported in constructed wetland systems where longer hydraulic retention times allow for greater pollutant removal [9]. Seasonal variations are also evident, with faster improvements observed during the July–September period. This may be attributed to enhanced root and vegetative growth during this period due to favourable weather conditions [21] and increased microbial activity, which enhances the biodegradation of organic matter and nutrient cycling processes [40,41].

Table 12 further evaluates sodium-related characteristics of the treated wastewater (Sodium Absorption Ratio-SAR). There is a progressive and significant (*p* < 0.05) reduction in values with increasing retention time. For example, during the March–May period, values decrease from 1.81 (raw) to 0.98 for both macrophytes after 21 days. Although the reduction trend is consistent, slight variations between *Phragmites karka* and *Typha latifolia* are observed. In some cases, *Typha latifolia* shows slightly lower values, suggesting a marginally higher efficiency in sodium reduction. However, both macrophytes effectively reduce sodium levels to ranges considered safe for irrigation use. The observed reductions can be attributed to multiple processes, including plant uptake, adsorption onto soil or sediment particles, and dilution effects. These processes collectively reduce the sodium hazard and improve the suitability of wastewater for agricultural applications. According to irrigation water quality guidelines, low sodium levels are essential for maintaining soil structure and crop productivity [11]. The SAR obtained for all treatments was ranked excellent according to the international standard (Supplementary Data; Table S9) [25].

Table 12 presents the Sodium Absorption Ratio (SAR) of poultry–aquaculture wastewater treated with *Phragmites karka* (PT) and *Typha latifolia* (TT) across different retention periods and seasons. SAR is an important indicator of irrigation water quality because it reflects the potential sodium hazard to soil structure and permeability. Elevated SAR values can lead to soil dispersion, reduced infiltration, and impaired crop productivity [26]. The results showed a progressive decline in SAR values with increasing retention time for both macrophyte treatments in all seasons. Initial SAR values in the raw wastewater ranged from 1.81 to 1.96, indicating the presence of appreciable sodium relative to calcium and magnesium ions. However, after treatment, SAR values decreased substantially, reaching minimum values of 0.99–1.34 after 21 days of retention. This trend demonstrates the effectiveness of the constructed wetland system in reducing sodium-related salinity hazards. The reduction in SAR may be attributed to several mechanisms, including plant uptake of sodium ions, ion exchange within the substrate, sedimentation processes, and microbial interactions within the rhizosphere. Macrophytes are known to facilitate ionic balance by absorbing excess sodium and promoting the retention of divalent cations such as calcium and magnesium in the treatment system [9]. The gradual decline observed with increasing retention period further indicates that longer hydraulic retention enhances contact between wastewater, substrate, plant roots, and microbial biofilms, thereby improving ion removal efficiency.

Seasonal variation was also evident in the SAR values. Slightly lower SAR values were recorded during the March–May period compared with November–January and July–September. This may be associated with increased biological activity and enhanced nutrient uptake during periods of favourable environmental conditions [42,43]. Nevertheless, the differences among seasons were relatively small, indicating stable treatment performance throughout the study period. Comparatively, *Typha latifolia* generally exhibited marginally lower SAR values than *Phragmites karka*, particularly at 21 days of retention during the November–January and March–May seasons. This suggests that *Typha latifolia* may possess a slightly greater capacity for sodium removal or ionic regulation. The differences may be linked to species-specific root morphology, growth characteristics, and rhizosphere interactions influencing ion uptake and transport. Investigating the seasonal impact on the heavy metal removal can be linked to climate change, which is a global phenomenon [44].

The superscript letters indicate significant differences (*p* < 0.05) among retention periods. Treatments at 21 days were significantly different from the raw wastewater, confirming that prolonged retention time substantially improved irrigation water quality. Importantly, all final SAR values remained below the critical threshold commonly associated with sodicity risk, indicating that the treated wastewater would be suitable for irrigation purposes without adverse effects on soil structure [26]. Overall, the findings demonstrate that both *Phragmites karka* and *Typha latifolia* effectively reduced sodium hazard in poultry–aquaculture wastewater, thereby enhancing its suitability for agricultural reuse within sustainable wastewater management systems.

### 3.7. Comparative Performance of Macrophytes, Environmental and Agricultural Implications Based on the Water Quality Assessment

Both *Phragmites karka* and *Typha latifolia* demonstrate significant improvement in water quality across all parameters. While the differences between the two systems are relatively small, *Typha latifolia* shows slightly better performance in reducing sodium percentage in some instances, whereas *Phragmites karka* exhibits comparable efficiency in improving overall WQI. The effectiveness of both macrophytes can be attributed to their extensive root systems, high biomass production, and ability to support diverse microbial communities. These characteristics enhance pollutant removal through physical filtration, biological degradation, and chemical transformation processes [9]. The results from Table 11 and Table 12 clearly demonstrate that untreated poultry–aquaculture wastewater is unsuitable for irrigation due to high pollution levels. However, treatment using macrophyte-based systems significantly improves water quality, making it suitable for agricultural reuse after sufficient retention time (21 days). The reduction in WQI and sodium-related parameters indicates that the treated wastewater can be safely used for irrigation without causing adverse effects on soil properties or crop yield. This highlights the potential of constructed wetlands as sustainable and cost-effective solutions for wastewater management in agricultural systems [45]. Overall, both macrophytes demonstrated high remediation efficiency; however, *Typha latifolia* showed slightly greater effectiveness in reducing sodium-related parameters and certain dissolved ions, while *Phragmites karka* exhibited comparable or marginally higher performance in organic matter removal (BOD_5_ and COD). These differences are attributed to variations in root architecture, biomass production, and rhizosphere activity, which influence microbial interactions and pollutant uptake pathways [31].

The results obtained in this study demonstrate that both *Phragmites karka* and *Typha latifolia* effectively reduced organic pollutants, nutrients, and heavy metals across varying seasonal conditions, indicating strong potential for scale-up in integrated poultry–aquaculture wastewater management systems. Constructed wetlands are widely recognised as sustainable, low-cost, and energy-efficient technologies that can be successfully adapted from pilot to field scale, particularly in developing regions where conventional wastewater treatment systems may be economically challenging. The extensive root systems, high biomass productivity, and pollutant tolerance of the studied macrophytes make them suitable candidates for long-term application in full-scale systems. However, we also acknowledge that large-scale implementation would require consideration of factors such as hydraulic loading rate, land availability, substrate management, climatic variability, and long-term biomass disposal. Therefore, while the present findings strongly support the feasibility of real-system application, additional long-term field-scale investigations are recommended to optimise operational conditions and evaluate economic sustainability.

### 3.8. Limitations and Recommendations for Future Research

The present study focused primarily on evaluating overall treatment efficiency through water quality improvement and multivariate analysis; therefore, quantification of heavy metals in plant tissues and the substrate was not considered in the present study. Such measurements would provide a more direct assessment of pollutant partitioning, and the above-mentioned measurement is out of the focus of this study. But it has now been indicated as a limitation and direction for future research. Nevertheless, based on the significant reduction in aqueous heavy metal concentrations and supported by established literature [31], it is well recognised that removal in constructed wetlands occurs through multiple pathways, including plant uptake, adsorption onto the substrate, and precipitation [31,35]. Consequently, it cannot be conclusively stated that metals were predominantly absorbed by plants or retained in the sand layer without direct mass-balance analysis. Since this is primarily out of scope for the present study. Its quantification based on mass balance analysis is suggested for further study.

In addition, the experimental design did not explicitly isolate or quantify the contributions of microorganisms or microalgae; however, their presence and role are inherently integrated within constructed wetland systems. It is well established that microbial communities in the rhizosphere and in biofilm attached to plant roots and substrate play a critical role in organic matter degradation, nutrient transformation, and metal immobilisation, while microalgae may contribute to nutrient uptake and oxygen dynamics [9,35]. Therefore, the observed treatment efficiency should be interpreted as a combined effect of plant–microbe–substrate interactions rather than plant activity alone. There is a need to separately quantify the impact of microbes on heavy metals in future research and studies.

## 4. Conclusions

This study evaluated the effectiveness of macrophyte-based phytoremediation systems using *Phragmites karka* and *Typha latifolia* for the treatment of poultry–aquaculture wastewater, with emphasis on physicochemical characteristics, heavy metal dynamics, and irrigation suitability. The results consistently demonstrated that both macrophytes significantly improved wastewater quality through integrated physical, chemical, and biological processes.

The correlation analyses revealed strong positive relationships among physicochemical parameters, such as turbidity, nutrients, biochemical oxygen demand (BOD_5_), and chemical oxygen demand (COD), indicating a common origin linked to organic pollution. Conversely, dissolved oxygen (DO) exhibited strong negative correlations with these parameters, reflecting oxygen depletion associated with microbial degradation of organic matter. Similarly, heavy metals showed strong positive interrelationships, suggesting shared anthropogenic sources and similar geochemical behaviour within the treatment systems. Principal Component Analysis (PCA) further confirmed that a few dominant factors—primarily organic pollution, nutrient enrichment, and metal interactions—accounted for most of the variability in the dataset. The high variance explained by the first few principal components indicates that wastewater quality is largely governed by a limited but significant set of environmental processes. Factor loadings and biplot interpretations revealed that both macrophytes effectively facilitated pollutant removal through mechanisms such as sedimentation, adsorption, plant uptake, and microbial transformation. The water quality assessment demonstrated substantial improvements with increasing retention time. The Water Quality Index (WQI) decreased drastically from extremely high values in raw wastewater to values below 1.0 after 21 days of treatment, indicating excellent water quality suitable for irrigation. Additionally, sodium-related indices showed consistent reductions, suggesting minimal risk of soil sodicity and confirming the suitability of the treated wastewater for agricultural reuse.

Comparatively, both *Phragmites karka* and *Typha latifolia* exhibited high treatment efficiency, with only minor differences in performance. *Typha latifolia* showed slightly better sodium reduction in some cases, while *Phragmites karka* demonstrated strong overall pollutant removal capacity. These differences may be attributed to variations in root structure, biomass production, and rhizosphere activity; however, both species proved highly effective for wastewater remediation. Overall, the findings highlight the potential of macrophyte-based constructed wetlands as sustainable, low-cost, and environmentally friendly technologies for treating poultry–aquaculture wastewater. The treated effluent, particularly after 21 days of retention, meets the criteria for safe irrigation use, thereby supporting water reuse in agriculture and contributing to sustainable water resource management. Future research should focus on long-term system performance, seasonal variability, and optimisation of design parameters to enhance treatment efficiency and scalability under different environmental conditions.

## Figures and Tables

**Table 1:** Methods/Instruments for wastewater characterisation.

Parameter	Parameter
Temperature	Thermometer (model, HI 9024 HANNA)
Turbidity	A nephelometer or turbidimeter was used for the determination of turbidity. The Hach Ratio Turbidimeter was used due to its general acceptability as well. The model of the instrument is WGZ-1B made by XINRUI Inc., **Shanghai,** China
Total hardness (TH)	Titration using ethylenediaminetetraacetic acid (EDTA)
BOD_5_	BOD_5_ self-check measurement (208215 OxiTop ^®^ IS 12, Global Water, a Xylem brand)
COD	Method 8000—reactor digestion method by a spectrophotometer, DR3900 model, made in Düsseldorf, Germany
Cl^−^	Silver nitrate titration method [20]
NO_3_^−^	Spectrophotometry (colourimetric method), coloured using 2 mL Nessler reagent and the colour change was spectrometrically read at a wavelength of 425 nm [22]
SO_4_^2−^	Gravimetric sulphate determination method [23]
Na, K, Ca, Mg	Atomic Adsorption Spectrophotometry (AAS)
As, Cd, Cu, Cr, Co, Fe, Pb, Mn, Zn, Ni	Atomic Adsorption Spectrophotometry (AAS)

**Source:** Akadiri et al. [21]. **Note:** BOD_5_ means the amount of Biochemical dissolved oxygen present in the wastewater samples before and after incubation in the dark at 20 °C for five days; COD is Chemical Oxygen Demand.

**Table 3:** Relationship between the physico-chemical properties of the poultry-aquaculture wastewater when treated and untreated with the *Phragmites karka* macrophyte plant.

Parameters	Colour	Turbidity	pH	DO	Cl^−^	NO_3_^−^	SO_4_^2−^	BOD_5_
Turbidity	0.984 *							
pH	0.964 *	0.951 *						
DO	−0.956 *	−0.974 *	−0.964 *					
Cl^−^	0.924 *	0.96 *	0.897 *	−0.958 *				
NO_3_^−^	0.951 *	0.972 *	0.96 *	−0.991 *	0.933 *			
SO_4_^2−^	0.965 *	0.981 *	0.975 *	−0.99 *	0.955 *	0.984 *		
BOD_5_	0.966 *	0.979 *	0.962 *	−0.991 *	0.953 *	0.991 *	0.983 *	
COD	0.968 *	0.98 *	0.964 *	−0.991 *	0.961 *	0.987 *	0.985 *	0.998 *

**Note:** * represents significance at 0.05.

**Table 4:** Relationship between the physico-chemical properties of the poultry-aquaculture wastewater when treated and untreated with the *Typha latifolia* macrophyte plant.

	Colour (HU)	Turbidity (NTU)	pH	DO	Cl^−^	NO_3_^−^	SO_4_^2−^	BOD_5_
Turbidity (NTU)	0.983 *							
pH	0.94 *	0.93 *						
DO	−0.949 *	−0.97 *	−0.956 *					
Cl^−^	0.92 *	0.959 *	0.864 *	−0.957 *				
NO_3_^−^	0.936 *	0.963 *	0.954 *	−0.993 *	0.939 *			
SO_4_^2−^	0.963 *	0.976 *	0.962 *	−0.988 *	0.948 *	0.98 *		
BOD_5_	0.958 *	0.971 *	0.95 *	−0.987 *	0.949 *	0.989 *	0.983 *	
COD	0.933 *	0.941 *	0.884 *	−0.955 *	0.948 *	0.949 *	0.946 *	0.974 *

**Note:** * represents significance at 0.05.

**Table 5:** Relationship between the heavy metals’ properties of the poultry-aquaculture wastewater when treated and untreated with the *Phragmites karka* macrophyte plant.

Parameter	As	Cd	Cu	Cr	Fe	Pb	Mn	Zn
Cd	0.909 *							
Cu	0.933 *	0.913 *						
Cr	0.947 *	0.956 *	0.966 *					
Fe	0.903 *	0.898 *	0.924 *	0.93 *				
Pb	0.951 *	0.904 *	0.972 *	0.963 *	0.921 *			
Mn	0.911 *	0.873 *	0.939 *	0.941 *	0.856 *	0.983 *		
Zn	0.956 *	0.902 *	0.96 *	0.981 *	0.952 *	0.971 *	0.944 *	
Ni	0.908 *	0.974 *	0.961 *	0.972 *	0.907 *	0.921 *	0.883 *	0.926 *

**Note:** * represents significance at 0.05.

**Table 6:** Relationship between the heavy metals’ properties of the poultry-aquaculture wastewater when treated and untreated with the *Typha latifolia* macrophyte plant.

Parameter	As	Cd	Cu	Cr	Fe	Pb	Mn	Zn
Cd	0.909 *							
Cu	0.933 *	0.913 *						
Cr	0.947 *	0.956 *	0.966 *					
Fe	0.903 *	0.898 *	0.924 *	0.93 *				
Pb	0.951 *	0.904 *	0.972 *	0.963 *	0.921 *			
Mn	0.911 *	0.873 *	0.939 *	0.941 *	0.856 *	0.983 *		
Zn	0.956 *	0.902 *	0.96 *	0.981 *	0.952 *	0.971 *	0.944 *	
Ni	0.908 *	0.974 *	0.961 *	0.972 *	0.907 *	0.921 *	0.883 *	0.926 *

**Note:** * represents significance at 0.05.

**Table 7:** Variance of the eigenvalues for the *Phragmites karka* and *Typha latifolia* macrophyte plants for the physico-chemical characterisation.

***Phragmites karka***									
Variance	3.0651	2.9688	2.8342	0.0892	0.0173	0.0108	0.0106	0.0031	0.0008
% Var	0.341	0.33	0.315	0.01	0.002	0.001	0.001	0	0
***Typha latifolia***									
Variance	3.1852	2.5148	1.6516	1.5587	0.0526	0.0216	0.0073	0.0065	0.0018
% Var	0.354	0.279	0.184	0.173	0.006	0.002	0.001	0.001	0.000

**Table 8:** (**a**): Factor loading of the physico-chemical properties characterisation using *Phragmites karka* macrophyte plant. (**b**): Factor loading of the physico-chemical properties characterisation using the *Typha latifolia* macrophyte plant.

(**a**)			
**Variable**	**Factor 1**	**Factor 2**	**Factor 3**
Colour	**0.523**	−0.464	**−0.714**
Turbidity	**0.609**	−0.495	**−0.611**
pH	0.429	−0.596	**−0.640**
DO	**−0.586**	0.633	0.495
Cl^−^	**0.780**	−0.440	−0.434
NO_3_^−^	**0.528**	**−0.682**	−0.501
SO_4_^2−^	**0.571**	**−0.588**	−0.546
BOD_5_	**0.573**	**−0.624**	−0.525
COD	**0.592**	**−0.599**	−0.530
(**b**)			
**Variable**	**Factor 1**	**Factor 2**	**Factor 3**
Colour	**0.547**	0.430	**−0.616**
Turbidity	**0.531**	0.550	**−0.536**
pH	**0.788**	0.357	−0.394
DO	**−0.634**	**−0.554**	0.348
Cl^−^	0.429	0.729	−0.380
NO_3_^−^	**0.653**	0.520	−0.321
SO_4_^2−^	**0.636**	0.520	−0.412
BOD_5_	**0.600**	0.500	−0.383
COD	0.455	0.519	−0.380

**Note:** Values greater than 0.5 were boldened with a positive sign in Factor 1 and a negative sign in Factors 2 and 3.

**Table 9:** Variance of the eigenvalues for the *Phragmites karka* and *Typha latifolia* macrophyte plants for the heavy metal characterisation.

				***Phragmites karka***					
Variance	3.0289	2.8342	1.9530	0.8968	0.2040	0.0765	0.0030	0.0025	0.0011
% Var	0.337	0.315	0.217	0.100	0.023	0.009	0.000	0.000	0.000
				***Typha latifolia***					
Variance	3.1031	3.0019	2.5739	0.2094	0.0560	0.0424	0.0099	0.0018	0.0014
% Var	0.345	0.334	0.286	0.023	0.006	0.005	0.001	0.000	0.000

**Table 10:** (**a**): Factor loading of the heavy metals’ properties characterisation using *Phragmites karka* macrophyte plant. (**b**): Factor loading of the heavy metals’ properties characterisation using **the** *Typha latifolia* macrophyte plant.

(**a**)			
**Variable**	**Factor 1**	**Factor 2**	**Factor 3**
As	**0.535**	−0.478	−0.413
Cd	0.433	**−0.769**	−0.379
Cu	**0.598**	**−0.532**	−0.442
Cr	**0.565**	**−0.604**	−0.436
Fe	0.429	−0.481	**−0.716**
Pb	**0.704**	−0.456	−0.437
Mn	**0.798**	−0.435	−0.333
Zn	**0.596**	−0.453	−0.535
Ni	0.453	**−0.730**	−0.392
(**b**)			
**Variable**	**Factor 1**	**Factor 2**	**Factor 3**
As	0.496	−0.544	−0.580
Cd	0.766	−0.450	−0.427
Cu	0.596	**−0.611**	−0.466
Cr	0.659	−0.517	−0.532
Fe	0.470	−0.417	**−0.771**
Pb	0.452	**−0.743**	−0.479
Mn	0.482	**−0.776**	−0.381
Zn	0.485	−0.568	**−0.631**
Ni	0.768	−0.460	−0.431

**Note:** Values greater than 0.5 were boldened with a positive sign in Factor 1 and a negative sign in Factors 2 and 3.

**Table 11:** Weighted average water quality index for poultry-aquaculture wastewater using the macrophyte plants.

Retention Days	November–December			March–May			July–September	
	PT	TT		PT	TT		PT	TT
Raw	4988.3 ± 224.2 ^a^	4988.3 ± 224.2 ^a^	Raw	3732.6 ± 125.1 ^a^	3732.6 ± 125.1 ^a^	Raw	3055.5 ± 98.1 ^a^	3055.5 ± 98.1 ^a^
7 days	3290.6 ± 123.1 ^ab^	3275.1 ± 118.2 ^ab^	7 days	2644.7 ± 74 ^ab^	32,753.4 ± 76.2 ^ab^	7 days	2445.4 ± 72.8 ^ab^	2265.3 ± 76.7 ^ab^
14 days	261.9 ± 8.2 ^c^	1666.1 ± 62.1 ^b^	14 days	1223.2 ± 58 ^b^	1594.1 ± 61.4 ^b^	14 days	7.53 ± 1.2 ^c^	7.27 ± 1.1 ^c^
21 days	0.43 ± 0.03 ^d^	0.37 ± 0.02 ^c^	21 days	0.41 ± 0.03 ^c^	0.461 ± 0.03 ^c^	21 days	0.39 ± 0.02 ^d^	0.39 ± 0.02 ^d^

**Note:** PT is *Phragmites karka*, and TT is *Typha latifolia;* means with different letters in the column are significantly different at 5% level significance using Tukey’s test. Significant differences are represented by lowercase letter superscripts. Data are presented as mean ± standard deviation.

**Table 12:** Sodium Absorption Ratio (SAR) of the poultry-aquaculture wastewater as affected by the macrophyte plants.

Retention Days	November–January		March–May		July–September	
	PT	TT	PT	TT	PT	TT
Raw	1.96 ± 0.06 ^a^	1.96 ± 0.06 ^a^	1.81 ± 0.05 ^a^	1.81 ± 0.05 ^a^	1.88 ± 0.06 ^a^	1.88 ± 0.06 ^a^
7day	1.82 ± 0.05 ^a^	1.81 ± 0.04 ^a^	1.40 ± 0.04 ^a^	1.38 ± 0.05 ^a^	1.66 ± 0.05 ^a^	1.66 ± 0.06 ^a^
14day	1.49 ± 0.04 ^ab^	1.44 ± 0.03 ^ab^	1.24 ± 0.03 ^ab^	1.21 ± 0.03 ^b^	1.38 ± 0.04 ^b^	1.40 ± 0.05 ^ab^
21day	1.10 ± 0.02 ^b^	0.99 ± 0.02 ^b^	0.98 ± 0.02 ^b^	0.98 ± 0.03 ^b^	1.18 ± 0.04 ^b^	1.34 ± 0.04 ^b^

**Note:** PT is *Phragmites karka*, and TT is *Typha latifolia*; means with different letters in the column are significantly different at 5% level significance using Tukey’s test. Significant differences are represented by lowercase letter superscripts. Data are presented as mean ± standard deviation.

## Data Availability

The data presented in this study are available on request from the corresponding author.

## Supplementary Materials

The following supporting information can be downloaded at: https://www.mdpi.com/article/10.3390/biology15110823/s1, Table S1: Initial Integrated poultry and Aquaculture Wastewater Characterisation (Physical and Chemical Properties); Table S2: Physical and chemical parameters of integrated poultry and aquaculture wastewater treated with *Phragmites karka* and *Typhia latifola* in a sub-surface constructed wetland at 7, 14 and 21 days retention periods during November 2021 to January 2022 season; Table S3: Physical and chemical parameters of integrated poultry and aquaculture wastewater treated with *Phragmites karka* and *Typhia latifola* in a sub-surface constructed wetland at 7, 14 and 21 days retention periods during March–May 2022 season; Table S4: Physical and chemical parameters of integrated poultry and aquaculture wastewater treated with *Phragmites karka* and *Typhia latifola* in a sub-surface constructed wetland at 7, 14 and 21 days retention periods during July–September 2022 season; Table S5: Heavy metal elements of integrated poultry and aquaculture wastewater treated with *Phragmites karka* and *Typhia latifola* in a sub-surface constructed wetland at 7, 14 and 21 days retention periods during November 2021 to January 2022 season; Table S6: Heavy metal elements of integrated poultry and aquaculture wastewater treated with *Phragmites karka* and *Typhia latifola* in a sub-surface constructed wetland at 7, 14 and 21 days retention periods during March 2022 to May 2022 season; Table S7: Some selected metals and heavy metal elements of integrated poultry and aquaculture wastewater treated with *Phragmites karka* and *Typhia latifola* in a sub-surface constructed wetland at 7, 14 and 21 days retention periods during July 2022 to September 2022 season; Table S8. Water quality status (WQS) based on the WQI; Table S9: Irrigation water classes according to the Sodium Absorption Ratio (SAR); Figure S1: Schematic representation of the vertical subsurface flow constructed wetland with nine replications; Figure S2: Set up of vertical subsurface constructed wetlands.
