# Supplementary material for: Comprehensive Evaluation of Vertical Sub-Surface Flow Constructed Wetlands with Aquatic Plants on Water Quality of Raw and Phyto-Remediated Poultry-Aquaculture Wastewater: A Principal Component Analysis
Source: Biology (Basel). 2026 May 23;15(11):823. doi: 10.3390/biology15110823 (PMC13255832; doi:10.3390/biology15110823)
Supplement: Supplementary file 1 [file biology-15-00823-s001.zip › biology-4308586-supplementary.pdf]

Table S1: **Initial Integrated poultry and Aquaculture Wastewater Characterization (Physical and Chemical Properties)**

Parameters	Unit	November 2021 – January 2022	March – May 2022	July – September 2022	National discharge standard	International discharge standard
		S1	S2	S3		
Appearance		DOC (C)	DOC (C)	DOC (C)	Clear	Clear
Odour		Obj	Obj	Obj	Unobjectional	Absent
Temperature	ºC	26.90	25.97	26.30	Ambient	Ambient
Colour	HU	11.70	12.80	11.50	5.15	5.13
Turbidity	NTU	10.24	12.50	12.00	10.00	10.00
Ph		8.31	8.25	8.10	6.50-8.50	6.50-8.50
DO	mg/L	0.10	0.18	0.23	4.00	6.40
Acidity	mg/L	131.00	125.00	115.00	-	-
Total Hardness (TH)	mg/$CaCO_3$/L	76.50	78.00	70.00	-	-
Cl^-	mg/L	48.50	52.00	60.50	250.00	350.00
NO_3^-	mg/L	39.70	42.10	38.70	40.00	50.00
SO_4^{2-}	mg/L	115.35	120.05	118.00	500.00	500.00
NH_3	mg/L	0.23	0.27	0.20	-	-
BOD_5	mg/L	28.50	32.10	27.90	6.00	4.00
COD	mg/L	54.80	61.50	53.70	30.00	30.00

Table S2: Physical and chemical parameters of integrated poultry and aquaculture wastewater treated with *Phragmites karka* and *Typhia latifola* in a sub-surface constructed wetland at 7, 14- and 21- days retention periods during November 2021 to January 2022 season

**Days**		7		14		21	
Water Treatment	RWW	PT	TT	PT	TT	PT	TT
Temperature	26.90[a]	27.04[a]	26.93[a]	26.57[b]	26.40[b]	26.37[b]	26.10[c]
Colour (HU)	11.0[a]	9.16[b]	9.11[b]	7.57[b]	7.60[b]	6.90[b]	7.08[b]
Turbidity (NTU)	10.24[a]	8.49[b]	8.29[b]	6.62[b]	6.93[b]	5.10[b]	5.41[b]
pH	8.31[a]	7.82[b]	7.95[b]	7.56[b]	7.69[b]	7.30[b]	7.37[b]
DO (mg/L)	0.10[b]	2.23[a]	2.18[a]	3.22[a]	3.21[a]	4.49[b]	4.73[a]
Acidity	131.0[a]	94.67[b]	95.11[b]	79.56[b]	72.78[c]	64.33[b]	64.00[b]
TA	197.0[a]	142.67[b]	138.67[b]	11.22[b]	105.67[c]	73.89[b]	67.11[c]
TH	76.50[a]	61.42[b]	61.03[b]	54.74[b]	54.99[b]	47.03[b]	48.11[b]
Cl- (mg/L)	48.50[a]	29.92[b]	30.56[b]	25.58[b]	25.16[b]	18.04[b]	18.47[b]
NO_3^- (mg/L)	39.70[a]	30.83[b]	29.57[b]	21.56[b]	21.07[b]	10.53[b]	9.73[b]
NO_2^- (mg/L)	0.30[a]	0.093[b]	0.092[b]	ND	ND	ND	ND
SO_4^{2-} (mg/L)	115.3[a]	79.77[b]	80.08[b]	63.89[b]	62.63[b]	33.40[b]	33.84[b]
BOD (mg/L)	28.50[a]	18.37[b]	19.07[b]	9.73[b]	9.36[b]	3.29[b]	2.48[b]
COD (mg/L)	54.80[a]	31.58[b]	29.96[b]	19.02[b]	1.39[b]	5.98[b]	4.66[b]
Na (mg/L)	77.56[a]	64.02[b]	63.00[b]	44.33[b]	43.03[b]	26.87[b]	23.92[c]
K (mg/L)	81.86[a]	70.35[b]	69.90[b]	49.81[b]	45.42[b]	31.44[b]	26.22[c]
Ca (mg/L)	42.74[a]	35.45[b]	33.48[c]	25.06[b]	23.76[b]	17.04[b]	16.41[b]
Mg (mg/L)	45.90[a]	35.68[b]	35.60[b]	25.42[b]	26.90[b]	17.32[b]	17.01[b]

Note: Means that do not share a letter are significantly different; RWW means Raw Wastewater; PT stands for *Phragmites karka;* and TT stands for *Typhia latifola;* ND means not detected

Table S3: Physical and chemical parameters of integrated poultry and aquaculture wastewater treated with *Phragmites karka* and *Typhia latifola* in a sub-surface constructed wetland at 7, 14- and 21- days retention periods during March – May 2022 season.

Days		7		14		21	
Water treatment	RWW	PT	TT	PT	TT	PT	TT
Temperature	25.90^{b}	26.14^{a}	26.12^{a}	26.72^{a}	26.18^{b}	26.12^{a}	26.03^{a}
Colour (HU)	12.80^{a}	8.36^{b}	8.42^{b}	7.7^{b}	7.8^{b}	5.70^{b}	5.70^{b}
Turbidity	12.50^{a}	8.17^{b}	8.18^{b}	6.91^{b}	6.86^{b}	4.54^{b}	4.42^{b}
pH	8.25^{a}	7.79^{b}	7.70^{b}	7.51^{b}	7.44^{b}	7.26^{b}	7.21^{b}
DO (mg/L)	0.18^{b}	2.12^{a}	2.17^{a}	3.36^{a}	3.47^{a}	4.88^{a}	4.94^{a}
Acidity	125.0^{a}	89.78^{b}	91.11^{b}	78.0^{b}	74.1^{c}	60.1^{b}	60.3^{b}
TA	187.0^{a}	127.33^{c}	130.89^{b}	100.2^{c}	108.3^{b}	8.33^{b}	79.89^{b}
TH	78.0^{a}	59.8^{b}	59.3^{b}	52.5^{b}	54.1^{b}	46.41^{b}	46.60^{b}
Cl- (mg/L)	52.0^{a}	34.7^{b}	33.7^{b}	27.5^{b}	27.1^{b}	20.01^{b}	18.20^{b}
NO_3^- (mg/L)	42.1^{a}	28.8^{b}	27.7^{b}	19.97^{b}	19.39^{b}	10.17^{b}	9.97^{b}
SO_4^{2-} (mg/L)	120.1^{a}	81.57^{b}	78.94^{c}	64.98^{b}	63.49^{b}	36.96^{b}	38.13^{b}
BOD (mg/L)	32.1^{a}	17.4^{b}	16.3^{b}	9.14^{b}	9.16^{b}	3.47^{b}	4.56^{b}
COD (mg/L)	61.5^{a}	32.3^{b}	30.6^{b}	17.6^{b}	17.5^{b}	6.69^{b}	5.96^{b}
Na (mg/L)	67.8^{a}	45.1^{b}	43.9^{b}	35.96^{b}	34.78^{b}	23.57^{b}	23.51^{b}
K (mg/L)	56.73^{a}	45.87^{b}	46.44^{b}	40.31^{b}	39.37^{b}	28.81^{b}	24.80^{c}
Ca (mg/L)	34.0^{a}	24.27^{b}	25.99^{b}	20.61^{b}	21.58^{b}	15.6^{b}	16.6^{b}
Mg (mg/L)	43.60^{a}	33.33^{b}	30.95^{b}	26.19^{b}	25.18^{b}	17.3^{b}	16.3^{b}

Note: Means that do not share a letter are significantly different; RWW means Raw Wastewater; PT stands for *Phragmites karka;* and TT stands for *Typhia latifola;* ND means not detected

Table S4: Physical and chemical parameters of integrated poultry and aquaculture wastewater treated with *Phragmites karka* and *Typhia latifola* in a sub-surface constructed wetland at 7, 14- and 21- days retention periods during July – September 2022 season.

Days		7		14		21	
Water treatment	RWW	PT	TT	PT	TT	PT	TT
Temperature	26.3[a]	26.1[a]	26.1[a]	26.99[a]	26.99[a]	25.99[b]	26.04[b]
Colour (HU)	11.50[a]	7.96[b]	7.86[b]	6.36[b]	6.43[b]	5.9[b]	6.08[b]
Turbidity (NTU)	12[a]	7.6[b]	7.5[b]	5.77[b]	6.03[b]	4.14[b]	4.3[b]
pH	8.1[a]	7.5[b]	7.75[b]	7.28[b]	7.21[b]	7.17[b]	7.12[b]
DO (mg/L)	0.23[b]	2.18[a]	2.29[a]	3.73[a]	3.72[a]	4.89[a]	4.73[a]
Acidity	115.0[a]	94.22[b]	92.89[b]	73.33[b]	72.78[b]	58.11[b]	58.67[b]
TH	70[a]	57.56[b]	57.33[b]	52.44[b]	51.56[b]	45[b]	45.33[b]
Cl- (mg/L)	60.5[a]	36.90[b]	36.10[b]	25.97[c]	28.49[b]	17.88[b]	18.50[b]
NO_3^- (mg/L)	38.70[a]	27.36[b]	29.04[b]	18.46[b]	18.41[b]	9.94[b]	8.81[b]
SO_4^{2-} (mg/L)	118[a]	74.68[b]	73.84[b]	42.53[b]	41.25[b]	29.37[b]	30.42[b]
BOD (mg/L)	27.90[a]	17.18[b]	16.51[b]	8.9[b]	8.73[b]	2.5[b]	2.33[b]
COD (mg/L)	53.70[a]	31.32[b]	30.91[b]	16.69[b]	16.49[b]	4.69[b]	4.29[b]
Na (mg/L)	65.90[a]	49.02[b]	48.24[b]	34.04[b]	33.7[b]	22.22[b]	24.41[b]
K (mg/L)	81.50[a]	57.04[b]	54.59[b]	41.59[b]	39.68[b]	26.64[b]	24.93[b]
Ca (mg/L)	29.50[a]	21.82[b]	22.43[b]	17.43[b]	17.73[b]	10.92[b]	11.42[b]
Mg (mg/L)	38.90[a]	27.10[b]	25.40[c]	17.41[a]	15.92[ab]	9.68[b]	8.26[b]

Note: Means that do not share a letter are significantly different; RWW means Raw Wastewater; PT stands for *Phragmites karka;* and TT stands for *Typhia latifola;* ND

Table S5: Heavy metal elements of integrated poultry and aquaculture wastewater treated with *Phragmites karka* and *Typhia latifola* in a sub-surface constructed wetland at 7, 14- and 21-days retention periods during November 2021 to January 2022 season.

Days		7		14		21	
Water treatment	RWW	PT	TT	PT	TT	PT	TT
As (mg/L)	0.10[a]	0.076[b]	0.074[b]	0.041[b]	0.031[c]	ND	ND
Cd (mg/L)	0.18[a]	0.119[b]	0.118[b]	0.0072[b]	0.061[b]	ND	ND
Cu (mg/L)	1.91[a]	1.45[b]	1.47[b]	1.01[b]	0.99[b]	0.27[b]	0.22[b]
Cr (mg/L)	0.28[a]	0.17[b]	0.20[b]	0.092[b]	0.085[b]	ND	ND
Co (mg/L)	0.199[a]	0.12[b]	0.11[c]	0.076[b]	0.076[b]	ND	ND
Fe (mg/L)	0.81[a]	0.507[b]	0.508[b]	0.25[b]	0.24[b]	0.13[b]	0.097[c]
Pb (mg/L)	0.21[a]	0.13[b]	0.14[b]	0.083[b]	0.083[b]	ND	ND
Mn (mg/L)	0.187[a]	0.127[c]	0.132[b]	0.082[b]	0.071[b]	ND	ND
Zn (mg/L)	2.94[a]	2.32[b]	2.28[c]	2.06[a]	1.99[a]	1.39[b]	1.22[b]
Ni (mg/L)	0.376[a]	0.239[b]	0.252[b]	0.103[b]	0.087[b]	ND	ND
Hg (mg/L)	0.012[a]	0.00178[b]	0.00156[b]	ND	ND	ND	ND
V (mg/L)	0.03[a]	0.0083[b]	0.0064[c]	ND	ND	ND	ND

Note: Means that do not share a letter are significantly different; RWW means Raw Wastewater; PT stands for *Phragmites karka;* and TT stands for *Typhia latifola;* ND means not detected

Table S6: Heavy metal elements of integrated poultry and aquaculture wastewater treated with *Phragmites karka* and *Typhia latifola* in a sub-surface constructed wetland at 7, 14- and 21-days retention periods during March 2022 to May 2022 season.

Days		7		14		21	
Water treatment	RWW	PT	TT	PT	TT	PT	TT
As (mg/L)	0.12^{a}	0.094^{b}	0.084^{b}	0.05^{b}	0.033^{c}	ND	ND
Cd (mg/L)	0.135^{a}	0.096^{b}	0.10^{b}	0.044^{b}	0.058^{b}	ND	ND
Cu (mg/L)	1.59^{a}	1.03^{b}	1.00^{b}	0.74^{b}	0.63^{c}	0.18^{b}	0.19^{b}
Cr (mg/L)	0.24^{a}	0.17^{b}	0.15^{b}	0.099^{b}	0.090^{b}	ND	ND
Co (mg/L)	0.16^{a}	0.11^{a}	0.199^{a}	0.073^{b}	0.058^{b}	ND	ND
Fe (mg/L)	0.94^{a}	0.39^{c}	0.46^{b}	0.22^{b}	0.26^{b}	0.14^{b}	0.17^{b}
Pb (mg/L)	0.19^{a}	0.12^{b}	0.10^{c}	0.075^{b}	0.056^{c}	ND	ND
Mn (mg/L)	0.16^{a}	0.12^{b}	0.11^{c}	0.077^{b}	0.081^{b}	ND	ND
Zn (mg/L)	3.10^{a}	2.36^{b}	2.36^{b}	2.00^{b}	1.89^{b}	1.37^{b}	1.47^{b}
Ni (mg/L)	0.26^{a}	0.17^{b}	0.18^{b}	0.092^{b}	0.099^{b}	ND	ND
Hg (mg/L)	ND	ND	ND	ND	ND		
V (mg/L)	ND	ND	ND	ND	ND	ND	ND

Note: Means that do not share a letter are significantly different; RWW means Raw Wastewater; PT stands for *Phragmites karka;* and TT stands for *Typhia latifola;* ND means not detected

Table S7: Some selected metals and heavy metal elements of integrated poultry and aquaculture wastewater treated with *Phragmites karka* and *Typhia latifola* in a sub-surface constructed wetland at 7, 14- and 21-days retention periods during July 2022 to September 2022 season.

Days		7		14		21	
Water treatment	RWW	PT	TT	PT	TT	PT	TT
As (mg/L)	0.12[a]	0.069[b]	0.064[b]	ND	ND	ND	ND
Cd (mg/L)	0.11[a]	0.089[b]	0.082[b]	ND	ND	ND	ND
Cu (mg/L)	1.84[a]	1.00[b]	1.02[b]	0.46[b]	0.40[b]	0.22[b]	0.16[b]
Cr (mg/L)	0.21[a]	0.13[b]	0.11[c]	0.059[b]	0.053[b]	ND	ND
Co (mg/L)	0.20[a]	0.098[b]	0.10[b]	0.055[b]	0.04[b]	ND	ND
Fe (mg/L)	0.77[a]	0.34[b]	0.28[c]	0.19[b]	0.19[b]	0.13[b]	0.15[b]
Pb (mg/L)	0.23[a]	0.14[b]	0.14[b]	0.055[b]	0.069[b]	ND	ND
Mn (mg/L)	0.20[a]	0.15[b]	0.15[b]	0.086[b]	0.069[c]	ND	ND
Zn (mg/L)	2.86[a]	2.16[b]	2.08[b]	1.81[b]	1.77[b]	1.3169[b]	1.22[b]
Ni (mg/L)	0.23[a]	0.15[b]	0.16[b]	ND	ND	ND	ND
Hg (mg/L)	ND	ND	ND	ND	ND	ND	ND
V (mg/L)	ND	ND	ND	ND	ND	ND	ND

Note: Means that do not share a letter are significantly different; RWW means Raw Wastewater; PT stands for *Phragmites karka;* and TT stands for *Typhia latifola;* ND means not detected

Table S8. Water quality status (WQS) based on the WQI.

WQI	WQS	Irrigation
0 - 25	Excellent	Suitable
25 - 50	Good	Suitable
50 - 75	Poor	Suitable
75 - 100	Very poor	Suitable
Above 100	Extremely Poor	Not Suitable

Source: FAO [11]
Note: WQI is water quality index, WQS is water quality status

Table S9: Irrigation water classes according to the Sodium Absorption Ratio (SAR)

SAR Value	Water suitability for irrigation	Sodium hazard class
SAR less than 10	Excellent	I
10 < SAR < 18	Good	II
18 < SAR < 26	Fair poor	III
SAR > 26	Unsuitable	IV

Note: SAR is Sodium Absorption Ratio (SAR);
Sources: Mustafa and Ali [25]

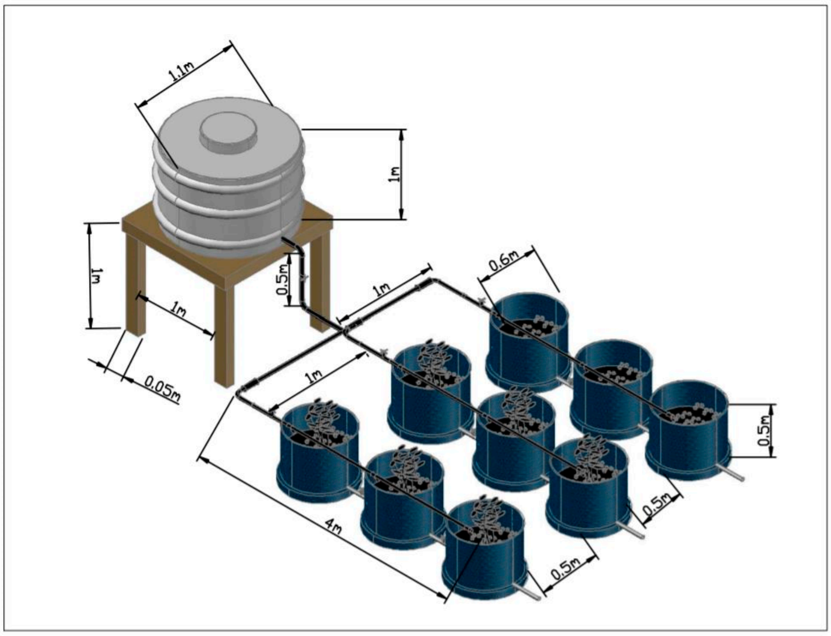

Figure S1: Schematic representation of the vertical subsurface flow constructed wetland with nine replications

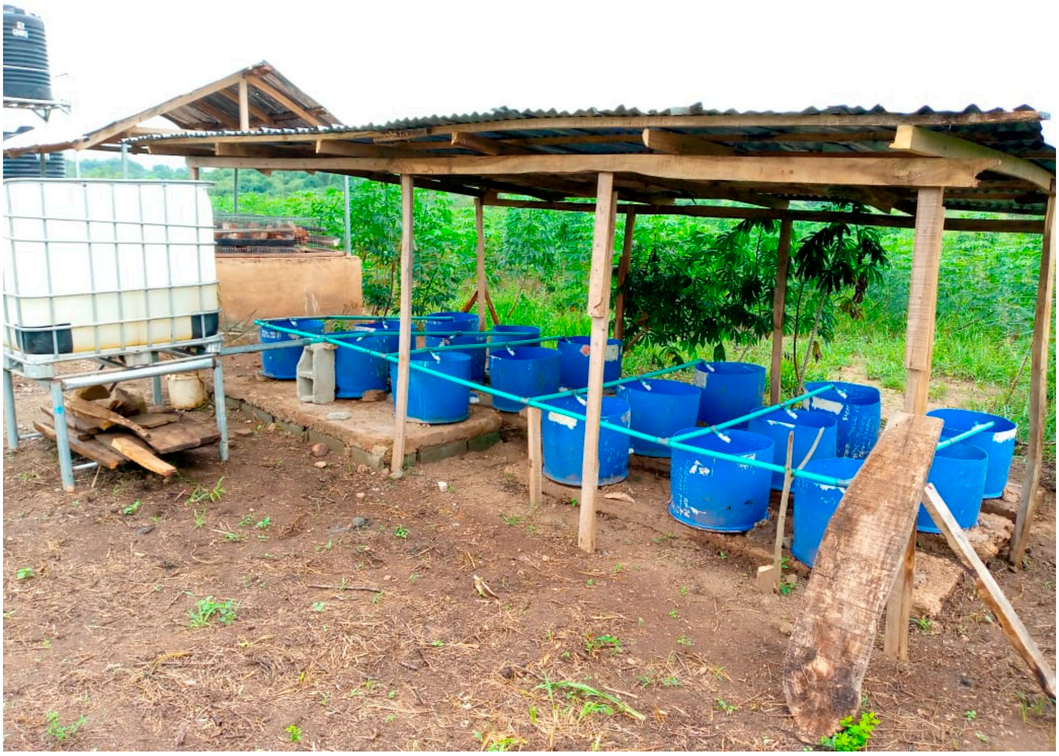

Figure S2: Set up of vertical subsurface constructed wetlands
